# Structural rearrangement of Neisseria meningitidis transferrin binding protein A (TbpA) prior to human transferrin protein (hTf) binding

**DOI:** 10.3906/kim-2102-25

**Published:** 2021-08-27

**Authors:** Gizem Nur DURAN, Mehmet ÖZBİL

**Affiliations:** 1 Department of Chemistry, Marmara University, İstanbul Turkey; 2 Institute of Biotechnology, Gebze Technical University, Kocaeli Turkey

**Keywords:** Neisseria meningitidis, transferrin binding protein TbpA, human transferrin protein hTf, molecular dynamics, structural rearrangement

## Abstract

Gram-negative bacterium Neisseria meningitidis, responsible for human infectious disease meningitis, acquires the iron (Fe^3+^) ion needed for its survival from human transferrin protein (hTf). For this transport, transferrin binding proteins TbpA and TbpB are facilitated by the bacterium. The transfer cannot occur without TbpA, while the absence of TbpB only slows down the transfer. Thus, understanding the TbpA-hTf binding at the atomic level is crucial for the fight against bacterial meningitis infections. In this study, atomistic level of mechanism for TbpA-hTf binding is elucidated through 100 ns long all-atom classical MD simulations on free (uncomplexed) TbpA. TbpA protein underwent conformational change from ‘open’ state to ‘closed’ state, where two loop domains, loops 5 and 8, were very close to each other. This state clearly cannot accommodate hTf in the cleft between these two loops. Moreover, the helix finger domain, which might play a critical role in Fe^3+^ ion uptake, also shifted downwards leading to unfavorable Tbp-hTf binding. Results of this study indicated that TbpA must switch between ‘closed’ state to ‘open’ state, where loops 5 and 8 are far from each other creating a cleft for hTf binding. The atomistic level of understanding to conformational switch is crucial for TbpA-hTf complex inhibition strategies. Drug candidates can be designed to prevent this conformational switch, keeping TbpA locked in ‘closed’ state.

## 1. Introduction

Neisseria is a gram-negative bacteria family, which was named after Albert Ludwig Sigesmund Neisser, a German physician who discovered the first species of the family, *Neisseria gonorrhoeae* in 1879 [1]. Among 11 members that can colonize in humans, only two of them are pathogenic: *Neisseria gonorrhoeae* and *Neisseria meningitidis* . *N. gonorrhoeae* invades urogenital tract causing gonoccal infection, whereas *N. meningitidis* invade nasopharynx, causing meningitis. Symptoms, such as fever, stiff neck, nausea, and vomiting, are observed within the first 24 h of infection in infants and older children. Even with appropriate treatment, 10%–15% of patients die; up to 21% of survivors can experience limb loss, chronic pain, and neurologic defects [2]. There are vaccines for *Neisseria* bacteria, however due to their limitations and the emergence of antibiotic resistant strains, there is a huge demand for more effective vaccine development [3,4]. *Neisseria* depends on iron (Fe) for its survival and virulence [5]. *Neisseria* doesn’t get the iron by making siderophore like other gram-negative bacteria. Instead, it directly abstracts iron from human transferrin (hTf) protein, which is facilitated by the neisserial transport system. This system includes two surface proteins, transferrin binding protein A (TbpA) and transferrin binding protein B (TbpB). TbpA is a ~100 kDa transmembrane protein, which is a member of TonB dependent transporters, requiring binding of TonB for internalization of the iron taken from the host [6]. TbpA is highly conserved among the *Neisseria* family [7] and forms TonB regulated β-barrel, where the abstracted iron can go through and reach the periplasm. On the other hand, TbpB is ~65–80 kDa lipidated protein bound to the outer bacterial membrane [8]. TbpA can bind and extract iron without TbpB present, although complexation with TbpB facilitates more efficient transfer [9]. Thus, TbpA is very important vaccine target. It is vital to understand how TbpA binds to hTf and how does two proteins interact with each other at the molecular level. 

The homology model of *N.meningitidis* TbpA protein by Oakhill et al. revealed that the β-barrel structure that sits in the outer trans-membrane region with hydrophobic amino acid residues lined on the outer surface and highly hydrophilic, charged amino acid residues forming the interior of the barrel [10]. This hydrophilic interior is believed to create a tunnel for acquired Fe^3+^ ion from hTf, which facilitates the transfer of the ion to ferric binding protein A (FbpA) located in the periplasm [11]. Other important feature of TbpA structure is the 158 amino acid long plug domain. The top of the domain creates a loop with short side-chain amino acids and is believed to interact with the hTf [10]. There are 11 external loops, whose lengths change from 4 to 85 amino acids [10,12]. These loops are located in the extracellular space, indicating their possible role in protein-protein interaction. In fact, Boulton et al. made deletions on loops 4, 5, and 8, and confirmed their primary role on hTf binding. Deletions of loops 4 and 5 made TbpA incapable of binding to hTf, whereas deletion of loop 8 created TbpA binding to hTf with approximately 10-fold decreased affinity compared to wild type TbpA [12]. In 2012, Noinaj et al. obtained X-ray structure of TbpA from *N. meningitidis* bound to only C-lobe containing hTf [13]. This structure revealed protein-protein interactions were facilitated by these extracellular loops and there is a charge complimentary between them and C-lobe hTf. Specifically, loop 5 created one long arm, and loops 7 and 8 created a shorter arm, which hug hTf from its C-lobe. There are 5 main TbpA regions creating intermolecular interactions with hTf. The 1st region is within loop 2 (sequence: D^251^SSNYA^256^), the 2nd region is within loop 3, which is also called ‘the helix finger’ (sequence: K^351^AVFDANKKQA^361^), the 3rd region is within loop 5 (sequence: Y^524^SSNTPPQNNGKKISPN^540^). Regions within loop 2 and loop 5 create the longer interaction arm. The 4th region, located within loop 8 (sequence: Y^708^EAQIKDGKEEAKGDP^723^) and the 5th region, located within loop 10 (sequence: R^825^ALLNGNSRN^834^) create the shorter interaction arms. 

Ferguson et al. previously proved experimentally that β-barrel domains of Ton-B dependent proteins such as ferric ion transport (FecA) protein undergo allosteric structural changes upon ligand binding [14]. This finding increases the possibility that TbpA might well undergo conformational changes upon binding to hTf. Moreover, the big cavity present in TbpA within two arms, long arm formed by loop 5, short arm formed by loop 8, might be smaller and less solvent exposed in the absence of binding partner. However, no such mechanism has been presented explicitly to this date. In the present study, we aimed to obtain hTf free TbpA structure. We identified structural mechanism preparing TbpA for hTf binding through classical all-atom molecular dynamics (MD) simulations. Conformational changes within hTf binding domains were analyzed, which will shed light on the complexation mechanism of transferrin binding proteins and ferric ion source proteins at the atomic level. 

## 2. Materials and methods

**Figure 1:**
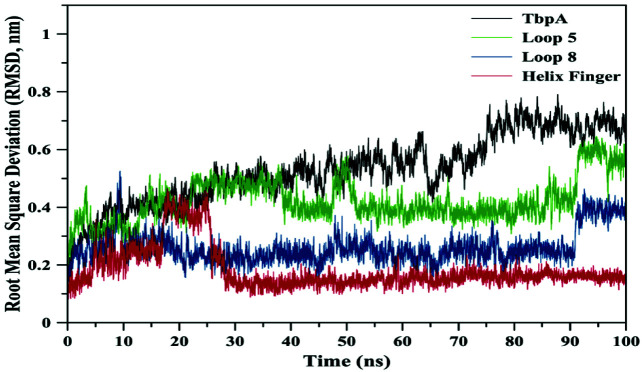
Root mean square deviation (RMSD) values for the whole TbpA protein, loops 5 and 8, and the helix finger.

The starting 3-D structure of TbpA was obtained from the X-ray structure of TbpA-hTf complex (PDB ID: 3V89) [13]. hTf protein and water molecules were stripped from the model. PROPKA server was utilized to calculate the protonation state of each titratable residue [15]. Molecular dynamics (MD) simulations were performed by GROMACS 5.1.4 software, [16–18] utilizing GROMOS 53A6 force field [19]. The transmembrane TbpA protein was embedded into DPPC (dipalmitoyl phosphatidylcholine) membrane model [20]. TbpA protein was placed into a cubic box with dimensions of 7.4 x 14.6 x 14.7 nm. These dimensions ensured that protein atoms stay in the simulation box throughout the simulation. The simulation box was filled with four-site interaction potential (TIP4P) for water molecules [21] and 30 sodium, 60 chloride ions were added to neutralize the system. First, starting system was subsequently energy-minimized using the steepest descent method for 50,000 steps. Then, energy-minimized structure was taken for the production phase. MD simulation without any constraints was carried out for 100 ns, with constant number of particles (N), pressure (P), and temperature (T), i.e. NPT ensemble. The SETTLE algorithm was employed to constrain the bond length and bond angle of the solvent molecules [22]. Bond lengths of the amino acid residues were constrained using LINCS algorithm [23]. Particle-mesh Ewald (PME) method was used to treat long-range electrostatic interactions [24]. A constant pressure of 1 bar was applied with a coupling constant of 1.0 ps and water molecules/ions were coupled separately to a bath at 323 K with a coupling constant of 0.1 ps. The equation of motion was integrated at 2 fs time steps using a leap-frog algorithm [25]. Production simulations were repeated twice, for the total simulation time of 200 ns. Tools available in the GROMACS and VMD 1.9.1 software [26] were utilized to analyze trajectories. Similar procedure and all-atom classical MD simulations were used in the literature to identify the motions of domains, regions of proteins [27]. The root mean square deviation (RMSD) graph on Figure 1 shows that the system has reached equilibrium in 70 ns by reaching out the plateau on the graph, although the RMSD value is high around 0.7 nm. Therefore, all the results and discussion were based on the data from the entire trajectory, instead of an average or energy minimum structure. 

## 3. Results

**Figure 2:**
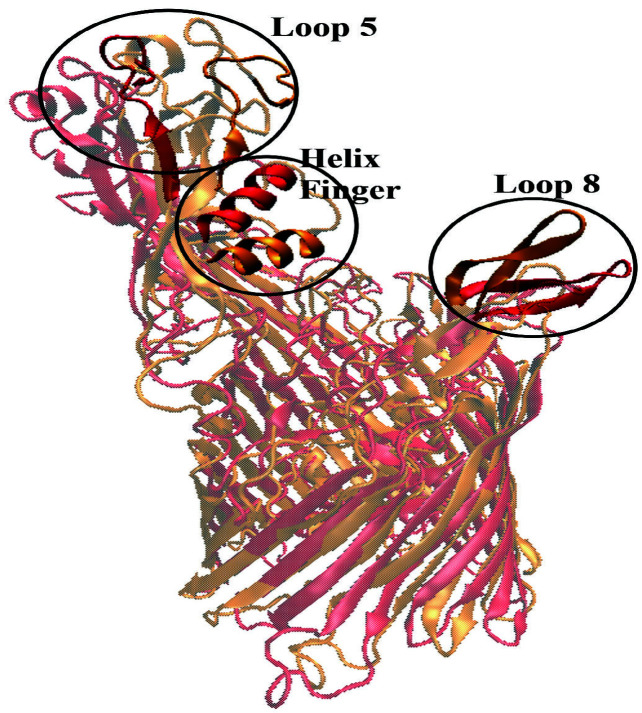
Alignment of TbpA X-ray structure (PDB ID: 3V89), in red, and the most representative TbpA structure from MD simulations, in orange. Three domains that play a critical role in hTf binding are circled and labeled. Conformational changes occurring in these domains will be discussed throughout the article.

Analysis of MD simulations highlighted significant conformational changes at three interface domains; namely loop 5, loop 8, and the helix finger. Structural alignment of the most representative TbpA protein structure from MD simulations and X-ray structure clearly revealed these three rearranged domains, which will be discussed in detail (Figure 2). 

### 3.1. Structural re-arrangement of loops 5 and 8

**Figure 3:**
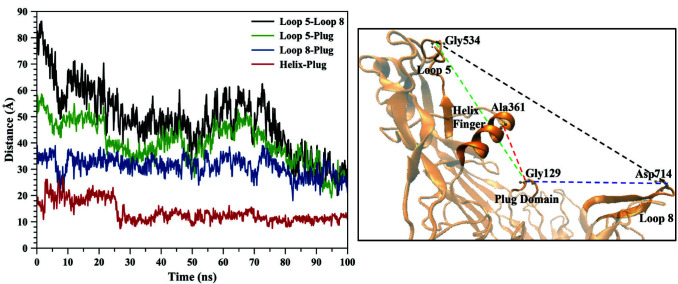
Distances between Cα atoms of loop 5-loop 8, loop 5-plug domain, loop 8-plug domain, and helix finger-plug domain were calculated from the entire 100 ns trajectory. Representations of each distance calculated were shown on the TbpA structure.

**Figure 4:**
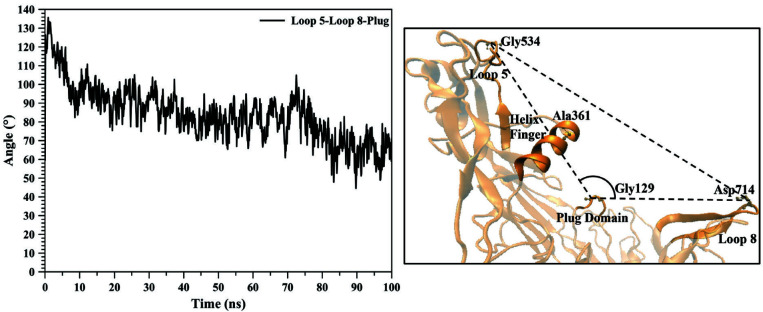
Angle between loops 5 and 8 and the plug domain, and loop 8 was calculated from the entire 100 ns trajectory. Representation of the angle calculated was shown on the TbpA structure.

As previously mentioned, loops 5 and 8 create two arms of TbpA, which hugs the hTf protein. Distance between two loops was measured from the TbpA-hTf complex X-ray structure. The reference atoms of the amino acids for this calculation were Cα of Gly 534 for loop 5 and Cα of Asp 714 for loop 8. The distance was measured as 79.81 Å. The distance shrinks throughout the MD simulation and the final value was 25.07Å. Figure 3 clearly reveals the continuous shrinkage until 95th ns and for the final 5th ns it remained same. The shrinkage could be due to the large movement of loop 5, loop8, and/or both loops combined. To identify whose movement contributes more, we set a reference atom that did not shift much during the simulation and measured the distance between loops 5 and 8 to that reference atom. The reference point was Cα of Gly 129 residue, which lies on the tip of the plug domain. Moreover, this atom remained in the middle of two loops in the entire MD simulation, which validates this choice as a reference point. Figure 3 indicated that loop 5 showed larger shift towards plug domain, whereas loop 8 showed moved less. Distance between Cα of Gly 534 for loop 5 and Cα of Gly 129 for plug domain was 50.53 Å in the X-ray structure. It continuously reduced and again after 95 ns it remained same around 26 Å (the final value was 25.88 Å). The distance between Cα of Asp 714 for loop 8 and Cα of Gly 129 for plug domain shrank less compared to loop 5-plug domain distance. Starting from 39.12 Å in the X-ray structure, it slowly shrank to 24.75 Å. Another measurement for analyzing the closing down of the two arms, was performed on the angle between loop 5, plug domain, and loop 8 (Figure 4). Here, similar to distance measurements, plug domain was used as a reference point, which helped to create the hinge angle between loops 5 and 8. The value of the angle in hTf bound ‘open’ state TbpA was 125.3° (X-ray structure). During the simulation it gradually decreased and after 80^th^ ns of the simulation it got stabilized around 60.0°. The shrinkage of this angle also clearly indicated that the two arms, loops 5 and 8, came closer to each other throughout the simulation transforming TbpA protein into a ‘closed’ state, where it can no longer bind to hTf protein (Figure 4). 

**Figure 5:**
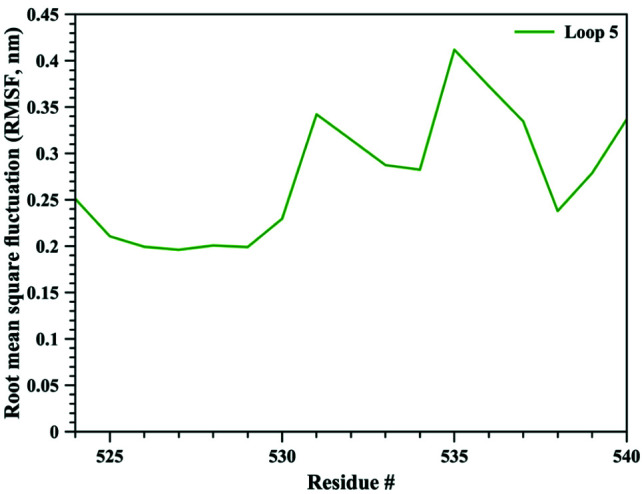
Root mean square fluctuation (RMSF) values for loop 5 residues.

**Figure 6:**
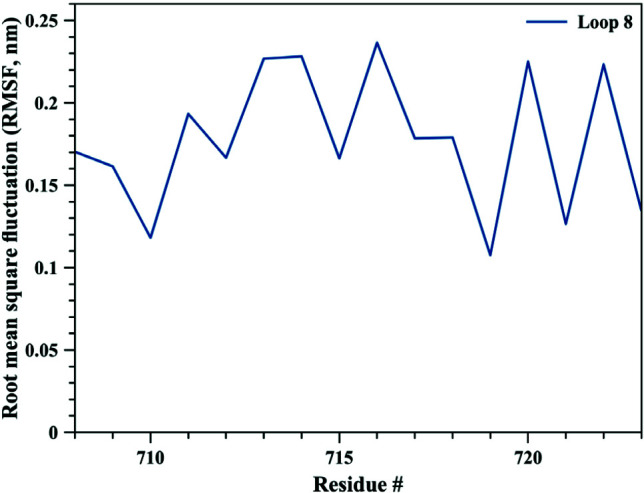
Root mean square fluctuation (RMSF) values for loop 8 residues.

**Figure 7:**
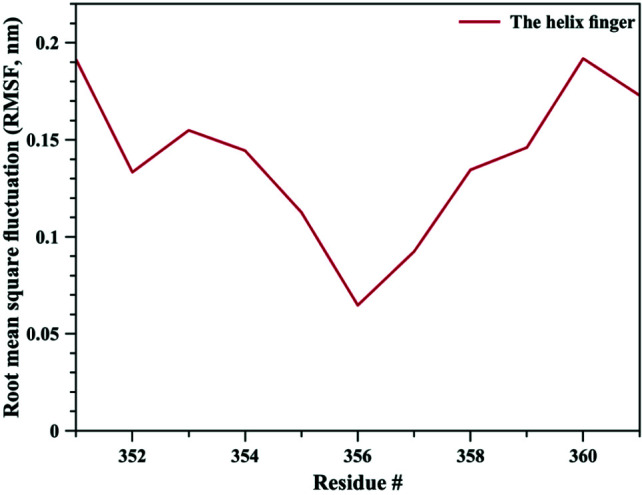
Root mean square fluctuation (RMSF) values for the helix finger residues.

The bigger shift and movement of loop 5 was also indicated by significantly higher RMSD values of loop 5 compared to those of loop 8. As it can be seen from Figure 1, RMSD values for loop 5 stayed around 0.4 nm whereas for loop 8 RMSD values were calculated around 0.25 nm. RMSD values for both loops jumped higher around 90th ns of the simulation, contributing the increase in RMSD values of the whole TbpA protein. These data clearly highlights that both loops 5 and 8 shifted towards each other, loop 5 contributing more to closing down of the arms. Root mean square fluctuation (RMSF) analysis, which is an indicator of fluctuation, in other words flexibility, of each residue clearly indicated that loop 5 became more flexible than loop 8 and the helix finger. Calculated RMSF values throughout MD simulation were between 0.20 nm and 0.42 nm for loop 5 (Figure 5), whereas RMSD values lied between 0.11 nm and 0.24 nm for loop 8 (Figure 6) and between 0.07 nm and 0.19 nm for the helix finger (Figure 7).

### 3.2. Structural re-arrangement of the helix finger: 

**Figure 8:**
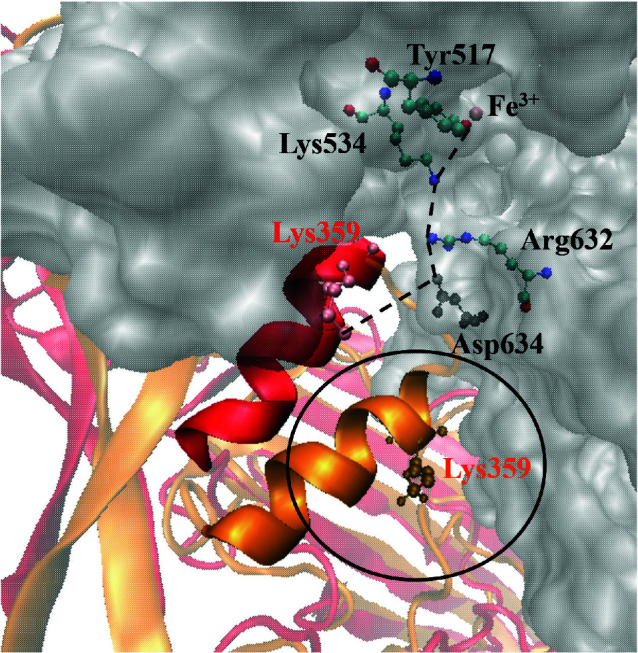
The proposed charge destabilization by Lys 359 of the helix finger in the X-ray structure (PDB ID:3V89). The H-bond network involved in stabilizing Fe^3+^ ion as shown with dotted lines and Lys 359, represented with red, is expected to disrupt this network, loosening the Fe^3+^ ion. Helix finger and Lys 359 in the most representative structure from MD simulations were shown in orange. Unfavorable clash with hTf (gray surface) and impossible interaction of Lys359 and Asp 634 was shown in black circle.

**Figure 9:**
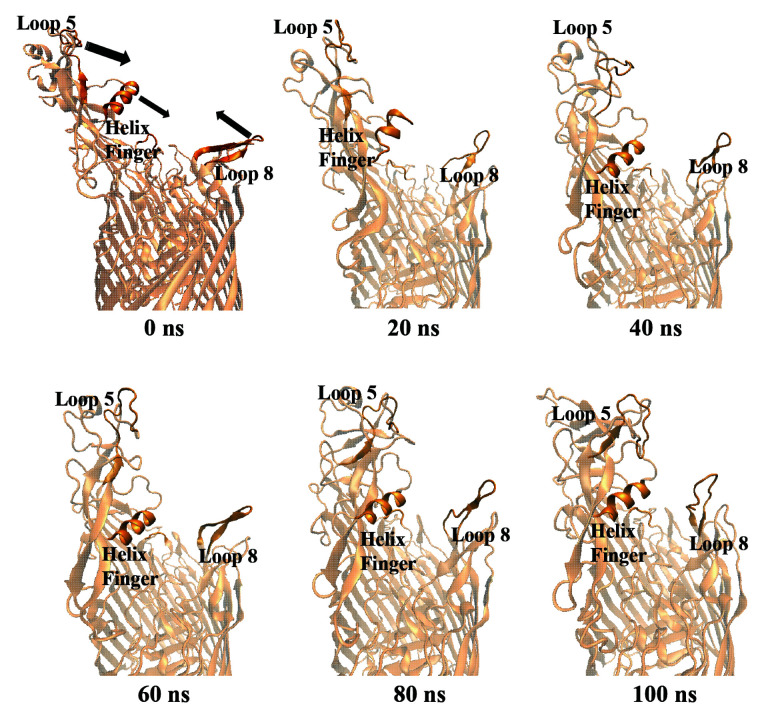
The 0th, 20th, 40th, 60th, 80th, and 100th ns snapshots of hTf binding domains of TbpA from MD simulations

The 2nd region, whose conformational change will be discussed, is within loop 3, which is also called ‘the helix finger’ (sequence: K^351^AVFDANKKQA^361^). In the X-ray structure of TbpA bound to hTf protein, this finger was reported to make close contact with C-terminal lobe of hTf [13]. Lys 359 residue of the helix finger was hypothesized to interact with Asp 634 residue of hTf and disrupt the salt bridges between Asp 634-Arg 632-Lys 534 amino acids. Disruption of these salt bridges will then disrupt the charge stabilization provided by hydrogen bonding between Arg 632 and Fe^3+^ binding Tyr 517. As a consequence, disruption of this hydrogen bond will lead to loosening of the Fe^3+^ ion and the bacteria can up take the ion (Figure 8). Thus, we have also analyzed the re-arrangement of the helix finger. The helix finger moved down throughout the simulation, coming closer to the plug domain. Figure 8 highlighted the downward movement of the helix finger, reaching the plug domain. Thus, this downward shift will also serve to the ‘closed’ state of TbpA protein. Once again, the plug domain was chosen as a reference point and the distance between Cα atom of Ala 361 for the helix finger and Cα atom of Gly 129 for the plug domain was measured (Figure 3). The distance was measured as 16.02 Å in the C-terminal hTf bound TbpA X-ray structure and at first 25 ns of the simulation it stayed above 20.0 Å. Between the 24th and 26th ns of the simulation, it got reduced drastically, bringing the helix finger towards the plug domain. Afterwards, the helix finger was stabilized (RMSD values on Figure 1 indicates this stabilization) and the distance remained stable on the range of ~11.0 to ~12.0 Å. It can be clearly seen on Figure 8 that, this new position of the helix finger would prevent it going deep into the C-terminal lobe of hTf and Lys 359 or any amino acid residues would not be able to disrupt the salt bridges mentioned above. Thus, TbpA protein was transformed into a ‘closed’ inactive state for hTf binding. Snapshots taken with 20 ns intervals also highlighted the conformational changes and drew a clear picture of the transformation from ‘open’ to ‘closed’ structure throughout the simulation (Figure 9). 

## 4. Discussion

*Neisseria meningitidis* bacterium up take Fe^3+^ ion from human host through triple complex comprised of bacterial TbpA, TbpB, and human hTf proteins. TbpA is a TonB-dependent receptor on the outer membrane that provides for iron to enter the cell. TbpA, which utilizes TonB-derived energy, is required for iron uptake from hTf [6]. TonB plays a role in the binding kinetics of hTf to TbpA, whereas which it does not directly affect TbpB binding. Upon disturbing TonB-TbpA interaction TbpA remains static and cannot carry out the iron transport. Once TbpA interacts with TonB, this receptor can carry out the remaining steps in hTf-iron acquisition [28]. The steps in TbpA iron acquisition comprise of TonB binding, hTf binding, removal of the iron, a change in conformation of the plug domain, transport of the iron across the outer membrane through TbpA, and release of the transferrin [29]. Although TonB binding was proposed the first step in TbpA mediated iron transfer, there is no experimental or theoretical data on the conformational change of TbpA upon binding. 

Isogenic mutants of TbpA and TbpB have been established to define the role of each protein in Fe^3+^ acquisition from hTf. The mutant bacterial complex lacking TbpA could not acquire Fe^3+^ from hTf, although it still was able to bind hTf. This confirmed that TbpA forms Fe^3+^ transport channel. On the other hand, TbpB was left alone, exposed to the surface, and it was able to bind hTf [30]. The iron transfer from the C-terminal lobe of hTf can proceed without TbpB at slow rate, but it cannot proceed in the absence of TbpA. Thus, TbpA binding to C-terminal lobe of hTf is considered as the most important step for the transfer. There are studies, mentioned above, proposing conformational changes on hTf upon TbpA binding. However, there is no study focusing on the conformational change of TbpA prior to the hTf binding, to our knowledge. In this study, we tried to identify structural changes that occur on free TbpA prior to hTf binding at the atomic level, by utilizing 100 ns long, all-atom MD simulations. When compared to hTf bound TbpA structure obtained from X-ray crystallography (PDB ID: 3V89), there were mainly three regions that underwent significant structural changes. These domains were the helix finger, loops 5 and 8, which create the hTf binding interfaces. The helix finger residue Lys 359 appears to interact with hTf residue Asp 634, which is one of three residues actively involved in the release of iron from the transferrin C-lobe [31]. Kinetics experiments with the stopped flow spectrofluorometer suggested that after Asp 634, a member of the C-lobe triad (Lys 534, Arg 632, and Asp 634), was mutated to alanine the rate of iron release increased ~83-fold. This result indicated that Asp 634 was needed to lock Fe^3+^ in the C-lobe hTF by neutralizing the positive charges of the other two amino acids in the triad, Lys 534 and Arg 632 [32]. Therefore the insertion of the TbpA helix finger upon complexation makes Lys 359-Asp 634 charge interaction plausible. This interaction causes charge repulsion between Lys 534 and Arg 632, thereby opening the iron binding cleft of the transferrin C-lobe and releasing Fe^3+^ [29]. These experimental data clearly highlights the importance of TbpA helix domain insertion. The helix domain shifted downwards significantly throughout MD simulations. The new position of this domain would make hTf binding impossible, as it would clash with hTf in the binding process. Moreover, loops 5 and 8, which create two arms of the protein that hug hTf in the crystal structure, shifted largely towards each other. Thus, the protein turned into a ‘closed’ state, in which hTf binding seemed impossible.

These conformational changes throughout the MD simulations, starting from hTf-bound X-ray structure of free TbpA protein, clearly revealed that apo TbpA protein remains in ‘closed’ state with loops 5 and 8 are closer to each other and the helix domain is more downwards. Prior to hTf binding, two loops must get away from each other in order to hug hTf from both sides. The helix finger domain swing up so that it can get into the cleft of hTf. Thus, upon TonB binding TbpA must get into the ‘open’ state prior to the complexation. The atomic level picture of conformational switch between ‘open’ and ‘closed’ states revealed in this study will play a critical role in the fight against meningococcal infections in the future experimental and theoretical studies. According to atomic picture revealed here, two regions might be targeted for small molecule conformational switch inhibitors. First region is between loop 5 and the helix finger domain. The 2nd region is between loop 8 and the helix finger domain. A small molecule inhibitor that will bind in one of these two regions or both at the same time might prevent the movement of loops 5 and 8 towards each other. Moreover, an inhibitor molecule fitting between loop 5 and the helix finger domain will also prevent the upward shift of the helix finger domain towards loop 5. Virtual screening of such small inhibitors targeting these two regions is underway. Supported with experimental studies discovering small inhibitors, to stop this conformational change may prevent TbpA-hTf binding will be crucial for the fight against meningococcal infections. 
